# Anatomy of the Teeth of Man and Animals

**Published:** 1839-08

**Authors:** Fr. Blandin, Harvey Burdell

**Affiliations:** Surgeon of the Hotel-Dieu, adjunct Professor of the Faculty of Medicine of Paris, etc.; Author of "Observations on the Anatomy, Structure, Physiology, and Diseases of the Teeth."


### ANATOMY OF THE TEETH OF MAN AND ANIMALS,

BY Ph. Fr. BLANDIN,

**Surgeon of the Hotel-Dieu, adjunct Professor of the Faculty of Medicine of Paris, etc.,--With Plates.**

*Translated from the French, with notes and general observations,*

BY HARVEY BURDELL, M. D.,

**Author of "Observations on the Anatomy, Structure, Physiology, and Diseases of the Teeth."**

*To the Editors of the American Journal of Dental Science.*

Gentlemen :—

The above is the title of a work on the teeth, that I have in course of translation, from which I take the liberty of sending you several extracts, which if deemed worthy of a place in your valuable Journal, you are at liberty to publish.

The translator is fully aware of the many disadvantages under which he labors, in being obliged to abridge many of the philosophical doctrinal positions taken by the author, in order to adapt them more directly to the limits of a Scientific Journal. He trusts however, that the brief extracts he shall furnish from time to time, will not be uninteresting to the practical dentist.

It is not the translator's intention to publish the whole of Blandin's work on the teeth in this form ; but only to present some of the important facts, and the new theories he has advanced, to the consideration of the members of the dental profession.

Professor Blandin has arranged his admirable and comprehensive treatise on the teeth, with great care, and in a very judicious manner. In the first place, he gives a general sketch of the history of the dental art, and of the advances made in this science from the earliest period to the present day ; he then gives an outline of the practical science of dental anatomy and dental surgery, and he also with much erudition describes the physical structure of the teeth of the human species. He concludes his labors by explaining the mode of development, arrangement, uses, &c., of these organs in various animals.

Harvey Burdell.

It will be necessary, (says the author) in giving the historical examination, to adopt some order of arrangement ; and I shall therefore, divide the historical portion of this work, into different periods, which will assist the reader in collecting other facts connected therewith. It will likewise be advantageous to myself, for it will furnish a kind of *halte*, by which I shall be assisted in these tedious and difficult researches.

### HISTORICAL SKETCH OF THE PROGRESS OF DENTAL SCIENCE.

—

### First Period.

*Of the progress of Dentistry in Egypt, China and Greece, to the time of Aristotle.*

According to Herodotus, the dental art, as well as the anatomy of the teeth was known to the early Egyptians, and the scientifiic pursuit of dentistry was followed by a certain sect, who had been educated to the profession.

In a report on the examination of an Egyptian mummy,* which M. Villeteau communicated to M. Sylvestre de Sacy, it is stated that the teeth were perfect, and not in the least affected by disease, although considerably worn away. It is also stated in this report, that even at the present day, the natives of Egypt have remarkably fine teeth, which are preserved to the extremest old age.

These are all the facts that I can gather in reference to the teeth and the history of Dentistry in Egypt, the cradle of the human sciences, and this I have been obliged to ask of silent tombs and mummified corpses.

In China, the science of anatomy has made but little progress, and the diseases and structure of the teeth are not studied. This is not surprising, when it is considered, that the Chinese never dissect the human subject; for they consider it an act of base cruelty to open a dead body, and they cannot even look upon human bones except with fright and horror! They however classify animals according to their external characteristics, and the teeth therefore, are of much service in the arrangement of the zoological divisions.

I have not been able to obtain much information, on the subject of the teeth, from the early history of Greece; for time, during the lapse of so many centuries, has destroyed almost every historical record that may have once existed; and all that I can find worthy of note, is in the philosophical definition given by the classic Homer, which is as follows:— The teeth says he, are *small barriers, placed in the mouth by nature, to prevent the tongue from going astray, or the abuse of words.* Eristratus however, mentions a tooth extractor, (odontagogue) made of lead, which was suspended in the temple of Apollo, in order, as it was said, to indicate

---

* Embalming bodies after death, was practised by the ancients centuries before and after Christ, from a belief that the vis vitæ or soul, returned after a certain period, to again inhabit the mortal tenement in a new and immortal capacity and to thus dwell where happiness and pleasure would be complete, among the beautiful and romantic fields of Elysium. H. B.

that the teeth should not be extracted, except when so loose that none but an instrument of lead need be employed in removing them.*

We must refer to the writings of the Greek philosophers for positive knowledge in reference to the teeth, and this we find among the writings of Hippocrates, or at least the views entertained respecting them.

Hippocrates says: "Frigidum inimicum ossibus, dentibus, nervis, cerebro, dorsali medullæ; calidum vero amicum." Several authors have concluded from the preceding statement, that Hippocrates distinguished the teeth from the bones; but is this understood or expressed in the foregoing quotation? It is more probable that Hippocrates merely wished to indicate, that the teeth, like the bones, are particularly injured from the effects of cold. He states, however, in another place, that the teeth are bones, or at least analagous to them.

He also states, that during the phenomena of dentition, much can be foretold, as to the future health of the individual, and that the teeth exhibit peculiarities in several diseases. He asserts positively, that the teeth or the rudiments of both sets, can be discovered in the fœtus. He speaks of the wisdom teeth, σωφρονιστηρες and agrees with other authors, that an extra number of teeth, is a sign of longevity, which is expressed in the rest of his text: Οι μαχρυσιοι πλεισους οδοντας εχουσι. He also said, that the irritation or inflammation in the teeth and gums of a female *enceinte*, was an evidence or symptom of super-fœtation.

---

### Second Period.

### *From Aristotle to Galen.*

Aristotle, that superior genius, was deservedly celebrated during his age and generation, on account of his knowledge of medicine and anatomy. He was the first who devoted himself to the study of the teeth, and he was likewise the first who duly considered them as peculiarly characteristic of the various classes of animals.

Among the writings of Aristotle, gross errors prevail, and many untenable theories are advanced; yet we nevertheless obtain in perusng them much valuable information. He wrote 350 years before the era of Christ. In order to trace the progress made in this department of science, from the age in which he lived down to the present period, it will not be out of place to give a general outline of the science at that time.

Aristotle says that the teeth present marked differences from man down to the lower order of animals; and that the different species of the latter, more particularly those called viviparous, have teeth as well as red blood, although the former do not exist equally in both jaws. Horned animals have no teeth in front of the upper jaw, but there are some

---

***In reference to this lead tooth extractor, much has been said and written. All the speculative controversies however, on this point, appear to have been based on the following quotation from an ancient classic work: "Erasistratus, plumbeum inquit odontagagum, quod nos dentiducum dicere poterimus, apud Delphum, in Appolinis Templo, ostentationes, causa propositum, quo demonstratur oportere cos dentes auferri, qui sint faciles, vel mobilitate laxati, vel quibus sufficiat plumbei ferramenti conamen ad summum." There is not sufficient evidence that such an instrument was ever exhibited in the temple of Apollo, as it has been pretended, and it is very probable that the preceding statement is founded on some fabulous story. H. B.**

that have neither upper front teeth nor horns, the camel for example.—Some animals have teeth projecting outward from the mouth, like the boar; others have them all pointed, like the lion, panther, dog, &c.; among others we see them flat and blunt, like those of the ox, horse, &c. No animal, it will be observed, has at the same time projecting teeth and horns, and not any of those that have teeth pointed, have either projecting teeth or horns. Aristotle calls all projecting teeth, *tusks similar to those of the Elephant.*

As a general rule says he, the teeth in front of the mouth are sharp and pointed, while the back ones are broader and wider. This rule, however, will not always apply, for the teeth of the seal are all sharp and pointed; hence this animal seems to connect quadrupeds with fishes.

Aristotle says, the teeth of the Elephant are their weapons for self-defence. He also says, that the front teeth are shed, in the human species; that the grinding teeth are not shed in man or animals; that the hog does not shed any of his teeth; that the age of many animals is known by their teeth; that the teeth become darker as age advances, except in the horse, whose teeth as they grow old become whiter.

Aristotle agrees with Hippocrates, that the appearance of more than the ordinary number of teeth is a presage of long life, and that those who have less than the usual number are generally shorter lived.

Men, says Aristole, have more teeth than women, and it is observed in some of the lower order of animals, that the males have more teeth than the females. It is a little surprising that such errors should have been promulgated by Aristotle, unless his texts have been altered, or as some suppose that he was only the historian, and recorded facts as well as fables, not being himself a practical anatomist.

He says the bills of birds are their teeth, and that they are analagous to the nails and horns, yet he contradicts his own argument in stating that the teeth are organized like the bones. * * * * To explain the only difference between teeth and the bills of birds, he says the former are shed, while the latter are not.

From Aristotle to Galen but little progress was made in the Dental art. It is however probable that Herophilus and Erasistratus, those two stars of the school of Alexandria, wrote on the subject of the teeth; but if such was the fact, their doctrines are lost, or at least have not reached us.*—It is said that Celsus wrote on the diseases of the teeth, and of the opera-

---

*Herophilus and Erasistratus, were the first systematic dissectors of the human subject The age in which they lived is not precisely known, but it is supposed to have been during the reign of Alexander the Great. Previous to the investigations of Herophilus and Erasistratus it was generally believed, that the amputation or removal of any material portion of the body, would prove fatal, but the experiments made by these *bold* surgeons changed the popular belief. We are informed that criminals after being condemned to death were handed over to Herophilus and Erasistratus by order of Alexander the Great, for them to try experiments upon, for the benefit of physical science, and they accordingly commenced their anatomical researches upon the living subject! The first experiments of this kind, were witnessed not only by the medical faculty, but by the Alexandrian Court and the Emperor himself. The operation consisted in cutting off an arm and from the circumstance that death did not immediately ensue, the operators and the whole court, fled from the scene, with the utmost fright and consternation, supposing the subject to be immortal! It was therefore ascertained from the investigations made by the school of Herophilus that the removal of a tooth, or even all of them at a single sitting would not prove fatal. H. B.

tions necessary to preserve them, and Archigeny it is said, was the first who recommended a small trepan to be applied in cases of violent tooth ache : it is therefore, probable that dentistry occupied the attention of scientific men at this period.

*Aretee* gives a specimen of his knowledge of the structure of the teeth, and concludes by asserting, that, " God alone knew the cause of tooth ache."

*Pliny*, has not progressed on the anatomy and structure of the teeth, any further than Aristotle. He has preserved the same errors, and relates many, more or less absurd stories respecting the teeth. He says, as an example of his fallacious theories, that the teeth of man contain a malignant virus, and that their bite might kill weak animals. He says also, that if two canine or eye teeth appear on the right side of the upper jaw, it is a presage of success and fortune. He relates a circumstance that occurred among the soldiers of the army of Germanicus Cæsar, during their campaign in Germany, which was, that the soldiers were in the habit of drinking sweet water from a certain fountain, and after following this practice for two years their teeth became loose and fell out. He cites a great many instances of the irregularity in the forms and structure of teeth, and quotes the story in reference to the teeth of Hercules, who it was said, had three sets of teeth; and likewise the fable of the sons of Prusias, (Curius and Papyrius,) who were born with teeth, and who were therefore, surnamed *dentati;* he speaks also of teeth developed in the palate, &c.

Aristotle says that all ruminating animals who have no projecting teeth in the front of their upper jaw, designed either for the purposes of attack or defence are supplied *by way of compensation*, with horns.

